# Cortical Tracking of the Speech Envelope in Logopenic Variant Primary Progressive Aphasia

**DOI:** 10.3389/fnhum.2020.597694

**Published:** 2021-01-06

**Authors:** Heather R. Dial, G. Nike Gnanateja, Rachel S. Tessmer, Maria Luisa Gorno-Tempini, Bharath Chandrasekaran, Maya L. Henry

**Affiliations:** ^1^Aphasia Research and Treatment Lab, Department of Speech, Language, and Hearing Sciences, University of Texas at Austin, Austin, TX, United States; ^2^SoundBrain Lab, Department of Communication Science and Disorders, University of Pittsburgh, Pittsburgh, PA, United States; ^3^Language Neurobiology Laboratory, Department of Neurology, Memory and Aging Center, University of California, San Francisco, San Francisco, CA, United States; ^4^Center for Neuroscience, University of Pittsburgh, Pittsburgh, PA, United States; ^5^Department of Neurology, Dell Medical School, University of Texas at Austin, Austin, TX, United States

**Keywords:** logopenic variant, logopenic variant of primary progressive aphasia (lvPPA), cortical tracking of speech, temporal response function (TRF), speech perception, speech envelope, speech envelope tracking

## Abstract

Logopenic variant primary progressive aphasia (lvPPA) is a neurodegenerative language disorder primarily characterized by impaired phonological processing. Sentence repetition and comprehension deficits are observed in lvPPA and linked to impaired phonological working memory, but recent evidence also implicates impaired speech perception. Currently, neural encoding of the speech envelope, which forms the scaffolding for perception, is not clearly understood in lvPPA. We leveraged recent analytical advances in electrophysiology to examine speech envelope encoding in lvPPA. We assessed cortical tracking of the speech envelope and in-task comprehension of two spoken narratives in individuals with lvPPA (*n* = 10) and age-matched (*n* = 10) controls. Despite markedly reduced narrative comprehension relative to controls, individuals with lvPPA had increased cortical tracking of the speech envelope in theta oscillations, which track low-level features (e.g., syllables), but not delta oscillations, which track speech units that unfold across a longer time scale (e.g., words, phrases, prosody). This neural signature was highly correlated across narratives. Results indicate an increased reliance on acoustic cues during speech encoding. This may reflect inefficient encoding of bottom-up speech cues, likely as a consequence of dysfunctional temporoparietal cortex.

## Introduction

Logopenic variant primary progressive aphasia (lvPPA) is a neurodegenerative language disorder characterized by a gradual dissolution of phonological processing ability. Individuals with lvPPA present with reduced phonological working memory capacity, impaired lexical retrieval and, in many cases, phonemic paraphasias in spontaneous speech. Cortical atrophy is most prominent in left temporoparietal cortex and spreads to additional regions subserving language and memory with disease (usually Alzheimer's) progression (Rohrer et al., 2010; Gorno-Tempini et al., 2011). Although phonological processing deficits are the defining feature of lvPPA, studies deconstructing and characterizing these deficits are lacking, especially in receptive language processing.

Receptive language processing at the single-word level is relatively intact in lvPPA but deteriorates as a function of stimulus length. This pattern has been attributed to phonological working memory deficits rather than semantic or syntactic processing impairments (Lukic et al., 2019). To date, processing of speech beyond single sentences has not been investigated in lvPPA. Further, functional neuroimaging studies investigating language processing in lvPPA have only examined processing of single syllables or words (Hardy et al., 2017). In everyday communication, however, we are rarely presented with single syllables, words or sentences in isolation, limiting the generalizability of previous research (Hamilton and Huth, 2018).

Recent analytical advances allow for online assessment of cortical tracking of ecologically valid, continuous speech stimuli, with the potential to generate findings that can be generalized to real-world situations. This approach involves fitting a temporal response function (TRF) to map speech features derived from naturalistic stimuli (e.g., audiobooks, movies) to temporally-precise neurophysiological data (Crosse et al., 2016). For example, TRF analyses can be used to assess the fidelity of cortical tracking of the speech envelope, which contains quasi-rhythmic amplitude fluctuations that are critical for transforming acoustic input into discrete representations (Ding and Simon, 2014), and the TRF time course can be used to infer the temporal dynamics of speech envelope encoding across the auditory processing hierarchy (Ding and Simon, 2012; Puvvada and Simon, 2017).

Cortical tracking of the speech envelope depends on neural oscillations in the delta (1–4 Hz) and theta (4–8 Hz) range (Ding and Simon, 2014), which have been linked to parsing linguistic structures from heard speech (Giraud and Poeppel, 2012). Delta oscillations are linked to parsing speech at the level of words and phrases as well as processing prosodic cues that are vital for constructing higher-level linguistic structures (Ding and Simon, 2014; Ding et al., 2017; Teoh et al., 2019); theta oscillations track the primary energetic rhythm in speech that is driven by low-level segmental features (syllables) (Ghitza, 2017). TRF analyses examining cortical tracking of the speech envelope by delta and theta oscillations have been applied to studies of continuous speech perception in neurotypical (Di Liberto et al., 2015) and clinical (Di Liberto et al., 2018; Fuglsang et al., 2020) populations.

Here we assess cortical tracking of the speech envelope of naturalistic, continuous speech in individuals with lvPPA (relative to age-matched controls) using EEG. This objective is motivated by converging evidence. First, neurodegeneration in lvPPA is most prominent in left temporoparietal regions, including the superior temporal gyrus and sulcus, middle temporal gyrus, and inferior parietal lobule (Rohrer et al., 2010), regions hypothesized to subserve the parsing of continuous speech input into perceptually-relevant units (Mesgarani et al., 2014). Second, aberrant neural processing of syllables presented in isolation has been observed in individuals with lvPPA (Hardy et al., 2017). Lastly, individuals with lvPPA show resting-state delta-theta hyperactivity in relatively spared frontal cortex as well as hypersynchrony in medial frontal and posterior parietal regions (Ranasinghe et al., 2017). We thus predict aberrant cortical tracking of the speech envelope in individuals with lvPPA relative to controls. This may manifest differently across oscillatory frequencies, so we assess the specificity (or lack thereof) across delta (word/phrase-level) and theta (syllable-level) oscillations in tracking of the speech envelope. Crucially, we evaluate the stability of this neural signature across two independent continuous speech narratives that vary in acoustic and linguistic complexity.

## Materials and Methods

### Participants

**Figure 1:**
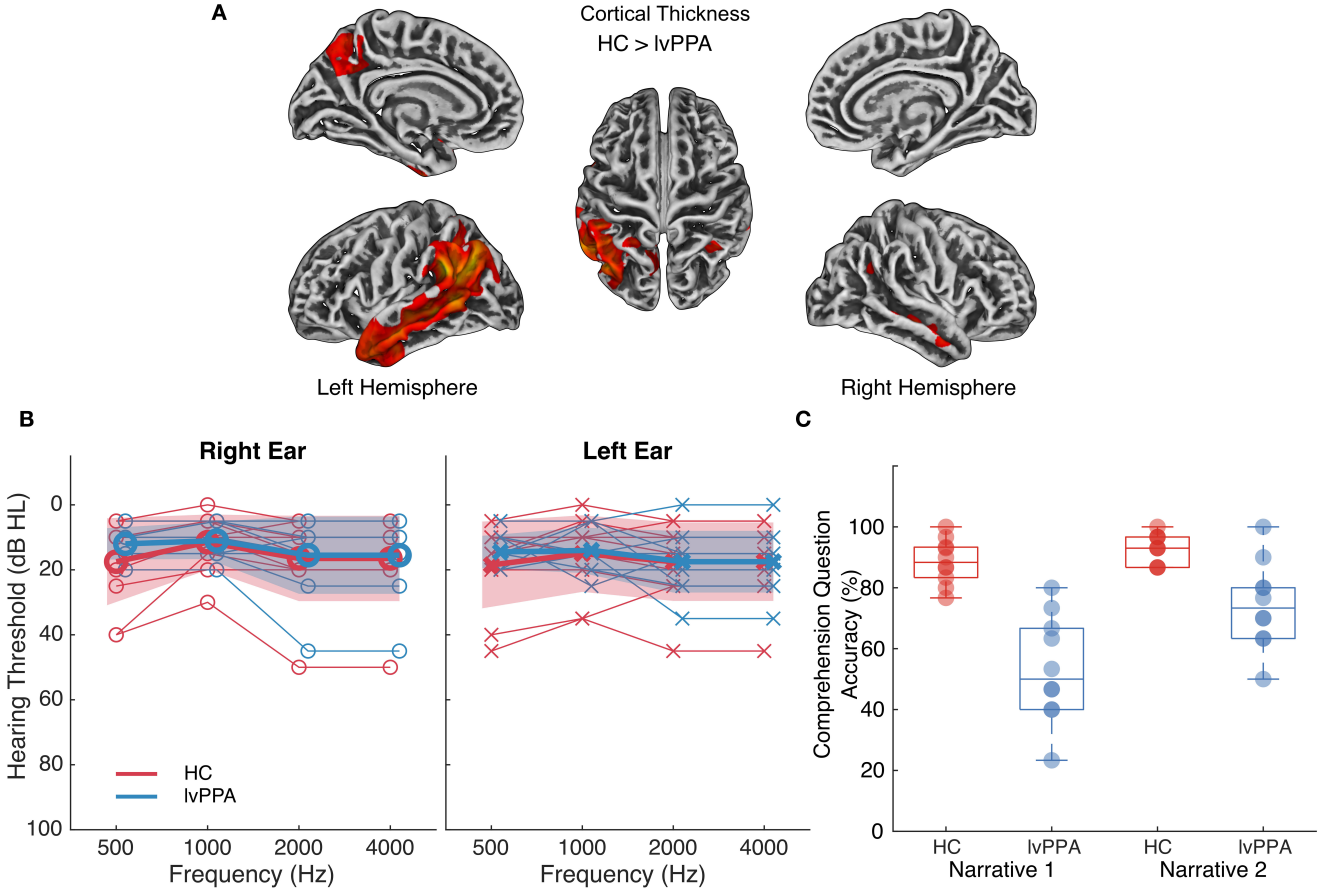
Atrophy profile, hearing thresholds and comprehension question accuracy for individuals with lvPPA relative to healthy controls. **(A)** Cortical thinning in individuals with lvPPA relative to *n* = 42 age-matched controls (two-sample *t*-test, controls > lvPPA, FWE-corrected *p* < 0.01, *k* = 133, covariates: age and sex). MRI scans were not available for HC presented in the current study, so a separate group of control data were used to identify significant cortical thinning in individuals with lvPPA. Lighter colors indicate more severe cortical thinning. **(B)** Pure tone hearing thresholds in healthy controls (HC) and individuals with lvPPA. Bold lines and markers represent group means. Thin lines and markers represent thresholds for each participant. The shaded area represents the standard deviation for the participant groups. Two (between-subjects, participant group [lvPPA, HC]) × 2 (within-subjects, ear [right, left]) × 4 (within-subjects, frequency [500, 1,000, 2,000, 4,000 Hz]) mixed ANOVA on hearing thresholds indicated that the two participant groups did not differ across frequencies or ears (two-way interactions with participant group and three-way interaction *p's* > 0.25). **(C)** Multiple-choice question accuracy (percent correct) in HC and individuals with lvPPA for each narrative.

Ten participants with a diagnosis of lvPPA (age *M* = 68.8, *s* = 8.8) and ten age-matched healthy controls (HC; age *M* = 65.9, *s* = 6.4) participated in the study. All participants were right-handed. Participants with lvPPA underwent comprehensive neurological and neuropsychological assessment and met current diagnostic criteria for lvPPA (Table 1); they had no history of stroke or other psychiatric, neurological, or medical diagnoses that could account for their language symptoms (Gorno-Tempini et al., 2011). Structural MRI scans showed significant cortical thinning in lvPPA participants localized primarily to left temporoparietal cortex, extending anteriorly within temporal cortex (Figure 1A). HC participants had no history of stroke, neurological disorders, or cognitive impairment. Hearing thresholds were assessed in all participants using pure tone audiometry (Figure 1B). All participants provided written informed consent. Study procedures were approved by the Institutional Review Board at the University of Texas at Austin and conformed to the Declaration of Helsinki.

**Table 1:** Performance on cognitive and linguistic assessments for individuals with lvPPA.

	**lv1**	**lv2**	**lv3**	**lv4**	**lv5**	**lv6**	**lv7**	**lv8**	**lv9**	**lv10**	**Mean (SD)**
Age (Years)	74	76	70	81	70	68	76	55	55	63	68.8 (8.8)
Sex^*^	M	M	F	F	M	M	F	F	M	F	–
Education (Years)^*^	16	20	12	18	18	16	16	18	18	13	16.5 (2.5)
Mini Mental State Examination (30)	19	26	22	18	27	23	26	26	23	27	23.7 (3.3)
Complex Figure Copy (17)^a^	15	16	11	15	17	17	15	17	17	15	15.5 (1.8)
Complex Figure Recall (17)^a^	6	10	2	7	14	10	12	14	7	9	9.1 (3.8)
Boston Naming Test (60)	33	44	34	9	45	40	56	10	33	50	35.4 (15.6)
Digit Span Forward^a^	4	5	6	5	3	5	5	4	4	4	4.5 (0.9)
Digit Span Backward^a^	3	4	2	3	3	3	4	3	3	3	3.1 (0.6)
WAB Aphasia Quotient	78.7	85.8	89.8	80.3	79.9	81.4	90.9	82.6	85.3	86.8	84.2 (4.2)
WAB Spontaneous Speech (20)	16	18	18	19	16	17	19	19	19	17	17.8 (1.2)
WAB Auditory Verbal Comprehension (200)	191	192	186	185	175	186	173	166	189	192	183.5 (9.0)
WAB Repetition (100)	74	80	94	67	61	68	82	78	62	80	74.6 (10.3)
WAB Naming and Word Finding (100)	64	73	82	52	91	76	96	62	80	88	76.4 (13.9)
Peabody Picture Vocabulary Test (16)^a^	15	16	16	14	16	15	16	13	15	13	14.9 (1.2)
Pyramids and Palm Trees: 3 pictures (60)	49	48	49	43	50	49	51	48	48	47	48.2 (2.2)
AOS Rating	0	0	0	0	1	0	0	0	0	0	0.1 (0.3)
Syntax Comprehension^b^	32/36	24/24	34/36	33/36	35/48	29/36	32/36	24/24	32/36	41/48	–
Northwestern Anagram Test (12)	9	10	11	9	1	9	7	9	10	11	8.6 (2.9)

*F, female; M, male; Inclusion criteria included Mini Mental State Examination (Folstein et al., 1975) scores of ≥ 15 (e.g., as in Henry et al., 2019). WAB, Western Aphasia Battery-Revised (Kertesz and Raven, 2007); AOS, Apraxia of Speech rating scale, where 0 = no impairment and 7 = profound impairment (Wertz et al., 1984); Boston Naming Test (Kaplan et al., 2001); Northwestern Anagram Test (Weintraub et al., 2009)*.

* *For controls, sex = 9F/1M and education M = 15.2, s = 2.3*.

a *Indicates assessments from neuropsychological battery described in Kramer et al. (2003)*.

b *The syntax comprehension task has 24 items per difficulty level, with two difficulty levels overall. Instructions indicate that the examiner should skip to the next level of difficulty if participants get the first 12 items correct for each difficulty level, so the total number of items tested can be 24, 36, or 48 (Wilson et al., 2010)*.

### Stimuli and Task

Stimuli consisted of two narratives (continuous speech) of ~15 min each: *Alice's Adventures in Wonderland* (narrative 1) (Carrol, 1865) and *Who Was Albert Einstein?* (narrative 2) (Brallier, 2002), the latter of which has been used and validated in stroke-induced aphasia (Wilson et al., 2018). Stimuli differed in acoustic (e.g., speech rate) and linguistic (e.g., clauses/sentence) features (see Supplementary Table 1 and Supplementary Figure 1 for details). The two narratives were segmented into 15 tracks of ~60 s each (narrative 1: 57–65 s; narrative 2: 56–67 s), with each track beginning and ending with a complete sentence, and presented binaurally using insert earphones (ER-3A; Etymotic Research, Elk Grove Village, IL). To assess narrative comprehension, after each track, participants were visually presented with two multiple-choice questions on a ViewPIXX monitor and selected one of four answer choices using a keyboard. A researcher assisted individuals with lvPPA by reading the questions aloud (if needed) and making keyboard responses.

### EEG Acquisition and Pre-processing

While participants listened to the narratives, EEG data were acquired using a 32-channel active electrode system (Brain Products, Gilching, Germany). Data were preprocessed using EEGLAB 2019.1 (Delorme and Makeig, 2004) in MATLAB 2016b (MathWorks Inc., Natick, MA, USA). Raw EEG data were downsampled to 128 Hz then filtered from 1 to 15 Hz. Following recommendations from de Cheveigné and Nelken (2019), we present the impulse and frequency response of the filter in Supplementary Figure 2. Channels with activity >3 standard deviations from surrounding channels were rejected and replaced via spherical spline interpolation. Artifact subspace reconstruction (ASR) was used to suppress large artifacts (Mullen et al., 2015). ASR-cleaned data were epoched from −5 to 70 s relative to stimulus onset and referenced to the common average of all channels. Independent component analysis was performed to correct for eye movement, muscle, and electrocardiographic artifacts. Given the proposed roles of delta and theta oscillations in relation to cortical tracking of speech (Ding and Simon, 2014; Ding et al., 2017; Ghitza, 2017; Teoh et al., 2019), the pre-processed signals were further bandpass filtered from 1 to 8 Hz, which we operationally defined as the full band. Lastly, since delta and theta oscillations may process different aspects of an incoming speech stream (i.e., words/phrases vs. syllables, respectively), the ICA-cleaned EEG data (1–15 Hz filtered) were furthered filtered into delta (1–4 Hz) and theta (4–8 Hz) bands to assess their different contributions to tracking of the speech envelope. Specific details regarding the filter parameters and additional analysis of data for different frequency bands are presented in the Supplementary Material.

### Cortical Tracking of Speech

**Figure 2:**
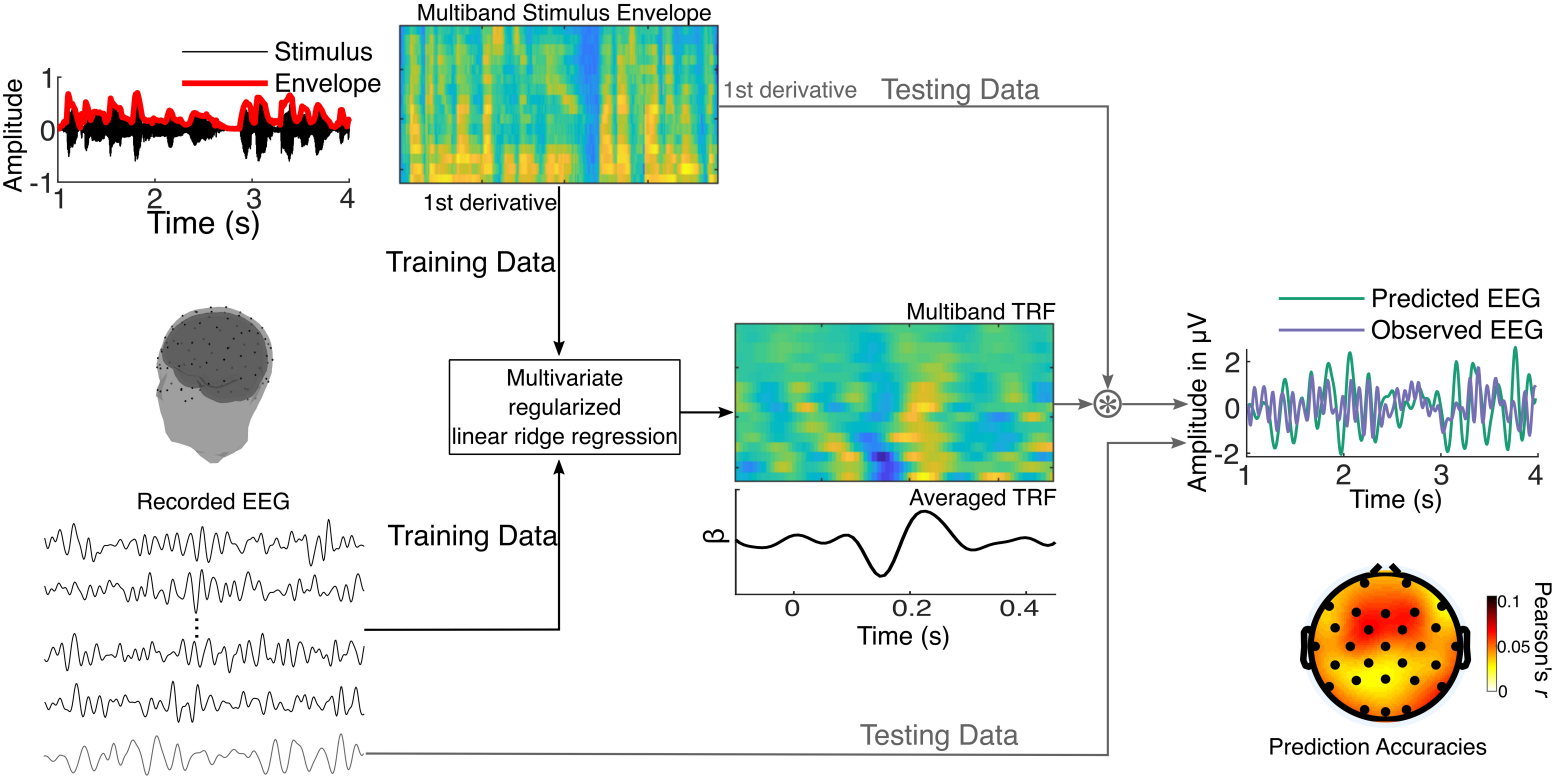
Schematic representation of the multivariate regularized linear ridge regression procedure used to obtain cortical tracking metrics. The stimulus envelope is extracted across sixteen gammatone filters resulting in a multiband stimulus envelope. This is used as the stimulus model and mapped onto recorded EEG using multivariate regularized linear ridge regression. In an iterative process, the temporal response function (TRF) is trained on a set of training data and tested on left-out testing data. Pearson's correlation coefficients (*r*-values) between the observed (testing) and predicted EEG data are obtained, reflecting the accuracy of the prediction. This is performed using leave-one-out cross-validation. The TRFs and prediction accuracies are estimated for each EEG channel and stimulus segment.

To evaluate the extent to which neural responses could be predicted by the speech envelope, the multiband stimulus envelope model was mapped onto preprocessed EEG data using a forward modeling TRF approach (Figure 2) implemented using regularized linear ridge regression (Lalor and Foxe, 2010; Crosse et al., 2016; McHaney et al., 2020; Reetzke et al., in press).

#### Multiband Speech Envelope Stimulus Model

To derive the multiband speech envelope, the auditory stimuli were first filtered using a bank of 16 gammatone filters uniformly spaced on an ERB scale from 250 to 8,000 Hz (Slaney, 1998). The absolute value of the Hilbert transform in each of the 16 bands comprised the multiband stimulus envelope, which was then raised to a power of 0.6 to mimic the compression characteristics of the inner ear (Vanthornhout et al., 2018). This resulted in 16 band-specific speech envelopes. The auditory cortex has been shown to be more sensitive to acoustic edges than sustained stimulus features (Hamilton et al., 2018), thus we derived the edges in the speech envelope using the first temporal derivative of the 16 band-specific envelopes. These edges signal rapid amplitude changes and predominantly represent onsets and offsets of acoustic events, such as phonemes and syllables.

#### TRF Estimation

The TRF estimation procedure is illustrated in Figure 2 and was done separately for the full (1–8 Hz), delta (1–4 Hz), and theta (4–8 Hz) bands. Each participant's EEG data were z-scored to the mean of all channels before TRF estimation. The TRF was estimated for each narrative by minimizing the least squares distance between EEG predicted from time-lagged features of the multiband speech envelope (−100–450 ms) and the observed EEG. Ridge regularization was used to smooth the TRFs and reduce overfitting, and the optimal ridge parameter was estimated for each participant. The resulting TRFs are a series of time-domain regression (beta) weights that explain the extent to which the EEG is predicted by the multiband speech envelope at different latencies. Prediction accuracy of the TRF was derived by obtaining Pearson's correlation coefficient (*r*) between the observed and TRF-predicted EEG. To reduce overfitting, the TRF was validated using leave-one-out (15-fold) cross-validation (Crosse et al., 2016), where the TRFs to *n* tracks were used to predict the EEG in the *k*th track. This cross-validation procedure was iterated to obtain the prediction accuracy for each track for each electrode. The prediction accuracies were then averaged across all the tracks and electrodes to get the final prediction accuracy, which we operationally defined as the cortical tracking metric. Lastly, to determine if the TRF is predicting the EEG at an above-chance level, we obtained the 97.5th percentile of the distribution of prediction accuracies for 1,000 permutations of mismatched stimulus track and EEG trials. All TRF analyses were performed using the mTRF_v1.4 Matlab toolbox (https://sourceforge.net/projects/aespa/files/; Crosse et al., 2016) and custom routines in Matlab (Mathworks Inc., Natick, Massachusetts).

Given the potential impact of EEG quality on the cortical tracking metrics, the signal to noise ratios (SNR) of the EEG recordings were obtained and used as a proxy to signal quality. The SNRs were estimated as a ratio of the root mean squares of post-stimulus-onset data (1.6–55 s after stimulus onset) and pre-stimulus-onset data (−2.6 to −1.6 s). The SNRs were then compared between the two groups for both narratives. A 2 × 2 mixed ANOVA (see Statistical Analysis section below) was used to compare SNR between the two narratives and the two groups. The main effects of narrative [*F*_(1,18)_ = 0.34, *p* = 0.570, $\eta_{G}^{2}$ = 0.002] and group [*F*_(1,18)_ = 1.56, *p* = 0.228, $\eta_{G}^{2}$ = 0.074] were not significant, nor was the interaction [*F*_(1,18)_ = 0.18, *p* = 0.674, $\eta_{G}^{2}$ < 0.001]. This ensured that the cortical tracking metrics were not biased by differences in signal quality between the two groups for the two narratives.

### Statistical Analysis

Multiple-choice question accuracy and cortical tracking metrics for each frequency band were analyzed via 2 (between-subjects, participant group [lvPPA vs. HC]) × 2 (within-subjects, narrative [narrative 1 vs. narrative 2]) mixed ANOVA using the afex package (version 0.26-0) (Singmann et al., 2020) in R (v3.6.3). Multiple-choice question accuracy (percentage) was adjusted using the rationalized arcsine transformation to mitigate ceiling effects (Studebaker, 1985). The data met assumptions for mixed ANOVA.

Whereas, the prediction accuracies quantify how well the multiband stimulus envelope is represented in the EEG, the TRFs inform the time-course of the neural dynamics underlying observed prediction accuracies. Thus, for frequency bands where significant differences were observed between lvPPA and HC participants, TRFs were compared between participant groups via 1,000 random, cluster-based permutations (Maris and Oostenveld, 2007) using Wilcoxon rank-sum statistics (two-tailed). The range for significant clusters is reported.

## Results

For multiple-choice questions, the main effects of participant group [*F*_(1,18)_ = 25.21, *p* < 0.0001, $\eta_{G}^{2}=$ 0.53] and narrative [*F*_(1,18)_ = 22.43, *p* < 0.001, $\eta_{G}^{2}=$ 0.20] were significant, as was the interaction [*F*_(1,18)_ = 7.17, *p* = 0.02, $\eta_{G}^{2}$ = 0.07]. HC performed similarly on the two narratives whereas individuals with lvPPA performed worse on narrative 1 (Figure 1C), although the effect size for the interaction was small.

**Figure 3:**
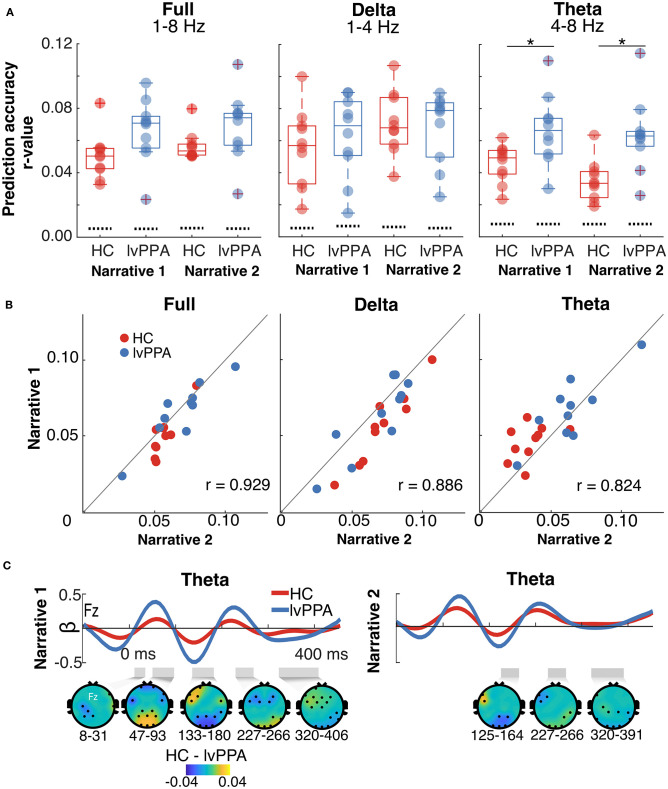
Cortical tracking metrics in healthy controls (HC) and individuals with logopenic variant primary progressive aphasia (lvPPA). **(A)** Prediction accuracies (Pearson's *r* averaged across all EEG channels) within full, delta, and theta bands for each narrative. The dotted lines show chance levels of prediction accuracies. The asterisk (*) highlights the significant main effect of group. **(B)** Prediction accuracies within full, delta, and theta bands for narrative 2 plotted as a function of narrative 1. Points lying on the unity line have equal prediction accuracies for the two narratives. The plots show that the prediction accuracies for both the narratives are highly correlated. **(C)** Frontocentral electrode (Fz) temporal response functions (TRF) for the theta band for each narrative. The gray shaded regions below each TRF mark the time regions that are significantly different between HC and lvPPA (significance derived via cluster-based permutation analyses). The scalp topographic plots below the TRFs show the difference (masked for significance) between the two groups.

All participants had above-chance-level prediction accuracies for full, delta, and theta bands (Figure 3A; see Supplementary Figure 4 for scalp distribution plots for the prediction accuracies). Prediction accuracies for both narratives were highly correlated (Figure 3B), indicating that this cortical tracking metric conveys reliable information about the encoding of the speech envelope irrespective of stimulus. For the full band, there was a significant main effect of narrative [*F*_(1,18)_ = 5.84, *p* = 0.03, $\eta_{G}^{2}$ = 0.02]. The magnitude of cortical tracking was significantly larger for narrative 1, although the effect size was small. The main effect of participant group [*F*_(1,18)_ = 3.80, *p* = 0.07, $\eta_{G}^{2}$ = 0.17] and the interaction [*F*_(1,18)_ = 1.10, *p* = 0.31, $\eta_{G}^{2}$ = 0.004], however, were not significant. For the delta band, the main effect of narrative was significant [*F*_(1,18)_ = 18.43, *p* < 0.001, $\eta_{G}^{2}$ = 0.05]; the magnitude of cortical tracking was significantly larger for narrative 2, although the effect size was small. The main effect of participant group [*F*_(1,18)_ = 0.04, *p* = 0.84, $\eta_{G}^{2}$ = 0.002] and the interaction [*F*_(1,18)_ = 4.01, *p* = 0.06, $\eta_{G}^{2}$ = 0.01] were not significant. For the theta band, the main effects of participant group [*F*_(1,18)_ = 10.75, *p* = 0.004, $\eta_{G}^{2}$ = 0.34] and narrative were significant [*F*_(1,18)_ = 5.75, *p* = 0.03, $\eta_{G}^{2}$ = 0.04], although the effect size for narrative was small. The main effects of participant group and narrative reflected a significantly larger magnitude of cortical tracking in the theta band for individuals with lvPPA and narrative 1, respectively. The interaction between participant group and narrative was not significant [*F*_(1,18)_ = 1.50, *p* = 0.24, $\eta_{G}^{2}$ = 0.01].

For the theta band, we examined the TRFs to characterize the time-course of the neural dynamics underlying observed prediction accuracies. Consistent with prediction accuracy results, individuals with lvPPA had significantly higher beta weights than HC in the theta band (narrative 1: 16.4 ≤ *z*_*clust*_ ≤ 71.8, narrative 2: 16.4 ≤ *z*_*clust*_ ≤ 21.24), indicating increased cortical tracking. This difference was seen throughout the time-course of the TRF with no specific difference at early or late latencies (Figure 3C).

## Discussion

Phonological processing impairment is the hallmark of logopenic variant primary progressive aphasia (lvPPA). This impairment has been examined using various tasks that broadly assess phonological processing (e.g., repetition, confrontation naming, sentence comprehension, phoneme manipulation), but research seeking to deconstruct this deficit and characterize the level(s) of breakdown is lacking. We assessed narrative comprehension and utilized temporal response function (TRF) modeling to examine cortical tracking of the speech envelope for two continuous speech narratives. Relative to healthy controls (HC), we observed reduced comprehension in lvPPA, extending previous findings of impaired comprehension for long sentences (Henry and Gorno-Tempini, 2010; Rohrer et al., 2010) using more ecologically valid, naturalistic, continuous speech stimuli. Cortical tracking metrics differed between lvPPA and HC, with significantly larger cortical tracking within the theta band (4–8 Hz) in lvPPA, but no difference between the two groups in the full (1–8 Hz) or delta bands (1–4 Hz). We also examined the TRF time-course, as latencies of TRFs have been associated with hierarchical cortical processing of auditory objects (i.e., latencies increase along the cortical processing hierarchy) (Ding and Simon, 2012; Puvvada and Simon, 2017). We observed larger regression coefficients (beta weights) for TRFs in the theta band in lvPPA over a broad time region, reflecting increased cortical tracking along the cortical processing hierarchy in both primary auditory and higher-level cortical regions. This neural signature was robust and consistent across two narratives that differed in acoustic and linguistic complexity, despite differences in comprehension for the two narratives. The findings of this study have implications for the characterization of deficits observed in lvPPA.

Temporoparietal cortex, which is significantly atrophic in lvPPA, plays a critical role in speech envelope encoding and the transformation of features represented in the envelope into discrete, perceptually-meaningful units (Mesgarani et al., 2014). Unlike stroke-induced aphasia, PPA is characterized by progressive degeneration of the left hemisphere language network (Rohrer et al., 2010). Longitudinal brain imaging in semantic variant PPA indicates that there is a gradual shifting in network dynamics in the context of atrophic changes (Canu et al., 2020). In fact, there is evidence of altered network connectivity in all three PPA variants (Agosta et al., 2014; Ranasinghe et al., 2017; Mandelli et al., 2018; Tao et al., 2020). Specifically in lvPPA (relative to HC), increased connectivity in right frontoparietal cortex has been observed using resting-state fMRI (Tao et al., 2020) and resting-state MEG has revealed bilateral changes within delta-theta oscillations, comprising both hypersynchrony between medial frontal and temporoparietal cortex and hyperactivity in the frontal cortex (Ranasinghe et al., 2017). Changes in network connectivity have been linked to changes in behavior inside (Wilson et al., 2009; Borghesani et al., 2020; Canu et al., 2020) and outside (Mandelli et al., 2018; Battistella et al., 2019) the MRI scanner in semantic and non-fluent variant PPA. In some cases, the recruitment of additional regions beyond sites of significant atrophy is related to better performance on speech and language tasks (Mandelli et al., 2018; Canu et al., 2020). Alternatively, some studies have found that the shift of function leads to errors (e.g., in the case of surface dyslexia in semantic variant PPA) (Wilson et al., 2009; Borghesani et al., 2020).

One possibility is that speech envelope encoding in individuals with lvPPA shifts away from atrophic temporoparietal cortex to relatively spared cortex that is not ideally suited to process information contained within the envelope. One might predict that this would lead to a *reduced* magnitude of cortical tracking, rather than an *increased* magnitude of cortical tracking. However, there are at least two candidate mechanisms underlying this paradoxical finding. First, increased cortical tracking of the envelope has been observed in the aging literature and argued to be a consequence of network degeneration and an imbalance between excitation and inhibition leading to inefficient information processing (Brodbeck et al., 2018). Second, the increased cortical tracking of the envelope may reflect functional hemispheric asymmetries. There is evidence that the cortical oscillations that support speech envelope encoding are lateralized, with faster oscillations being left lateralized (i.e., gamma oscillations) and slower oscillations (i.e., delta-theta oscillations) being right lateralized (Gross et al., 2013). Because left temporoparietal cortex is significantly atrophic in lvPPA, there may be an increased reliance on the right hemisphere for speech envelope encoding, thereby leading to increased cortical tracking by slower oscillatory frequencies, such as the theta band. The spatial resolution of EEG is relatively poor, and future research seeking to disentangle potential mechanisms (“what and where”) underlying the neural signature observed in this study will benefit from the use of tools such as MEG and source-level analyses.

In sum, we posit that increased cortical tracking of the speech envelope in individuals with lvPPA relative to HC is likely a consequence of shifting of function away from atrophic temporoparietal cortex to relatively spared brain regions in both hemispheres. Of note, this does not occur at a global level but rather, selectively enhances tracking of the syllabic rhythm of speech (4–8 Hz), as evidenced by increased cortical tracking in the theta band only. Phonological processing deficits in developmental dyslexia are argued to be a consequence of reduced envelope tracking within the theta range, leading to an increased sensitivity to phonetic features (Goswami, 2011). Our findings in lvPPA may represent the opposite pattern—increased reliance on syllabic structure during envelope encoding as a consequence of impaired processing of phonetic features due to left temporoparietal atrophy. Evidence for aberrant neural processing of phonetic features has been observed in left temporoparietal cortex in lvPPA (Hardy et al., 2017), lending some support to this theory.

Cortical tracking metrics, like those used in the current study, may have potential for use as a neural signature to inform differential diagnosis in PPA. To determine the viability of using such an approach for differential diagnosis, future research will require examination of our findings in lvPPA relative to other PPA variants and related disorders (e.g., Alzheimer's dementia, posterior cortical atrophy) to determine whether this neural signature is specific to lvPPA. Moreover, these metrics may provide valuable insights into perceptual deficits in PPA in naturalistic listening conditions that are not easily assessed using conventional clinical approaches. In conclusion, the current study points to an increased reliance on the speech envelope during continuous speech perception in lvPPA, arising from inefficient processing of acoustic cues within the theta band. The current study thus marks an important step toward more precise characterization of speech processing deficits in lvPPA.

## Data Availability Statement

The raw data supporting the conclusions of this article will be made available by the authors, without undue reservation.

## Ethics Statement

The studies involving human participants were reviewed and approved by the Institutional Review Board at the University of Texas at Austin. The patients/participants provided their written informed consent to participate in this study.

## Author Contributions

Data collection, analysis and background literature review were conducted by HD, GNG, and RT. Clinical diagnosis and characterization was conducted by HD, MG-T, and MH. Data interpretation and manuscript preparation were conducted by HD, GNG, RT, MG-T, BC, and MH. All authors contributed to the article and approved the submitted version.

## Conflict of Interest

The authors declare that the research was conducted in the absence of any commercial or financial relationships that could be construed as a potential conflict of interest.
