# Supplementary material for: Cortical Tracking of the Speech Envelope in Logopenic Variant Primary Progressive Aphasia
Source: Front Hum Neurosci. 2021 Jan 6;14:597694. doi: 10.3389/fnhum.2020.597694 (PMC7815818; doi:10.3389/fnhum.2020.597694)
Supplement: Supplementary file 1 [file Data_Sheet_1.DOCX]

Supplementary Material

**Supplementary Methods**

**Stimuli and Task**

Stimuli consisted of two narratives (continuous speech) of approximately 15 minutes each: the public domain audiobook *Alice’s Adventures in Wonderland* (narrative 1; [(Carrol, 1865)](https://www.zotero.org/google-docs/?7g2WTH)) and *Who Was Albert Einstein?* (narrative 2; [(Brallier, 2002)](https://www.zotero.org/google-docs/?R35fyz)). The narratives differed in measures of readability, lexical frequency, semantic and syntactic complexity, and words per minute (Supplementary Table 1). Each narrative was read in American English by a male speaker and sampled at a frequency of 22,050 Hz. The modulation spectrum of both narratives showed a peak between 4-8 Hz (Supplementary Fig. 1), which corresponds to the theta range of EEG oscillations. Narrative 2 had a slightly higher modulation magnitude at relatively lower frequencies compared to narrative 1.

Supplementary Table 1. Acoustic and linguistic characteristics of the two narratives used in the study.

| Measure | Narrative 1 | Narrative 2 |
| --- | --- | --- |
| Flesch Kincaid Reading Ease | 81.3 | 73.5 |
| Flesch Kincaid Grade Level | 6.8 | 5.8 |
| Gunning Fog Score | 9.2 | 7.5 |
| SMOG Index | 5.1 | 5.8 |
| Total Number of Words | 3650 | 2243 |
| Average Number of Words/Sentence | 19.84 | 11.16 |
| Clauses/Sentence^a^ | 2.71 | 1.54 |
| Average Frequency/Million Words^b^ | 6790.86 | 4822.99 |
| Complex Words/Total Words | 3.51 | 8.34 |
| Average Number of Syllables/Word | 1.25 | 1.44 |
| Average Number of Words/Minute | 240.61 | 146.60 |
| Bigram (slope)^c^ | -2.63 | -2.29 |

*Notes:* SMOG = Simple Measure of Gobbledygook.

^a^Clauses/sentence was computed for the first ~2000 words (if the 2000th word was in the middle of a sentence, that sentence was removed entirely from the transcript) for each text using the L2 Syntactic Complexity Analyzer (Lu, 2010; Lu, 2011; Ai & Lu, 2013; Lu & Ai, 2015).

^b^Average frequency/million computed as the average across all words. Each word’s frequency was obtained using the Corpus of Contemporary American English database for ALL sections (Davies, 2019).

^c^Bigrams were calculated using the bag of words function in Matlab. Bigrams convey semantic meaning along with other lexical and syntactic information (Mitchell, 2013).


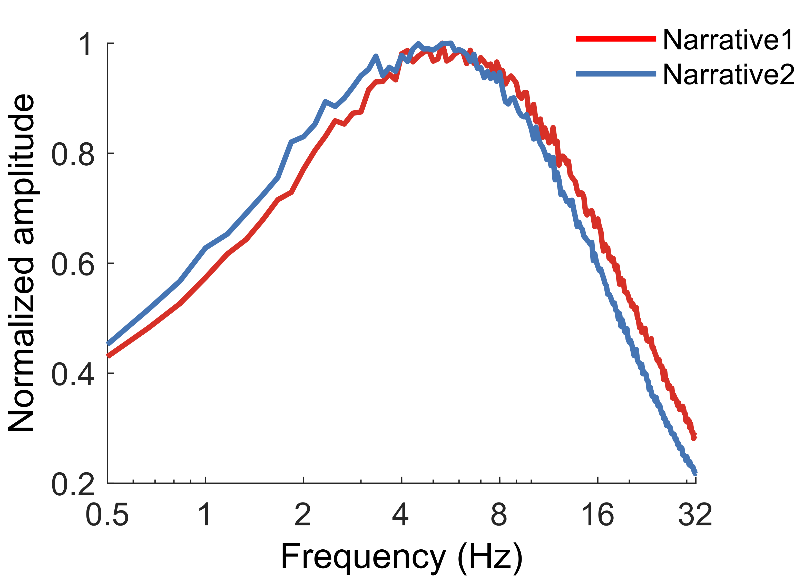


Supplementary Figure 1: Mean modulation spectrum (based on Ding et al., [(2017)](https://www.zotero.org/google-docs/?rNT9fa)) of the two narratives used in the current study. Narrative 2 had slightly more energy concentration at lower modulation frequencies than narrative 1. Both narratives show peak modulation between 4-8 Hz.

Auditory stimuli were presented binaurally using insert earphones (ER-3A; Etymotic Research, Elk Grove Village, IL). First, participants were presented with two one-minute tracks of a different story (*Sherlock Holmes*), which served as practice trials and allowed us to adjust the volume to a comfortable listening level on an individual basis. To minimize eye movements while each track played, a fixation cross was presented in the middle of a VIEWPixx monitor placed directly in front of the participant. After each track, participants were visually presented with two multiple-choice questions on the monitor and required to select one of four answer choices using a keyboard. Following the second multiple-choice question, participants pressed a button to proceed to the next track when they were ready. For individuals with lvPPA, a researcher sat in the sound-dampened EEG room and assisted by reading the multiple-choice questions aloud (if needed) and making keyboard responses. Participants were instructed to remain as still as possible while listening to each track but could move while answering questions. After the practice trials, participants listened to 15 tracks of narrative 1, followed by a short break and 15 tracks of narrative 2 following the same procedure. Stimuli were presented in E-Prime 2.0.10 software [(Schneider *et al.*, 2002)](https://www.zotero.org/google-docs/?nRMTLX).

**EEG preprocessing**

EEG data were preprocessed using EEGLAB 2019.1 [(Delorme and Makeig, 2004)](https://www.zotero.org/google-docs/?XYMmLn) in MATLAB 2016b (MathWorks Inc., Natick, Massachusetts, USA). Preprocessing and analysis were not blinded by participant and was done by the first and second authors. Raw EEG data were downsampled to 128 Hz. Downsampled data were filtered from 1 to 15 Hz, consistent with previous cortical tracking research. A non-causal, Hamming windowed-sinc FIR filter was used for bandpass filtering (high pass filter cut-off = 1 Hz, filter order = 846; low pass filter cut-off = 15 Hz, filter order = 212). The filter characteristics are shown in Supplementary Fig. 2 based on the recommendations of de Cheveignѐ and Nelken [(2019)](https://www.zotero.org/google-docs/?TcdtGq). Subsequently, channels with activity more than 3 standard deviations from the surrounding channels were rejected and replaced via spherical spline interpolation; for four control participants, one channel was interpolated, whereas no channels were interpolated for lvPPA participants. Artifact subspace reconstruction (ASR) was used to suppress large artifacts [(Mullen *et al.*, 2015)](https://www.zotero.org/google-docs/?6fQjJB). Approximately 60 seconds of clean data were manually identified and input as the calibration data for ASR. ASR-cleaned data were epoched from -5 to 70 seconds relative to stimulus onset. The epoched data were re-referenced to the common average reference and independent component analysis (ICA) was performed to correct eye-movement, muscle and electrocardiographic artifacts. ICA was performed using infomax algorithm runica.m, adjusted to extract 25 components in order to meet full rank order assumptions of ICA. Components were removed based on manual inspection of time-course, topography and spectrum. For lvPPA participants, an average of 3.5 components was removed for narrative 1 (range 2 – 5), and 3.8 components for narrative 2 (range 2 – 6). For HC, an average of 3.5 components was removed for narrative 1 (range 2 – 5), and 3.5 components for narrative 2 (range 2 – 5).





Supplementary Figure 2. Impulse and magnitude responses of the EEG filters used to separate the EEG bands. In blue are the characteristics of the high pass filter and in yellow are the characteristics of the low pass filter.

The ICA-cleaned EEG data (1-15 Hz filtered data) were furthered filtered using the non-causal, Hamming windowed-sinc FIR filter into full (1-8 Hz; low pass filter cut-off = 1 Hz, order = 846; high pass filter cut-off = 8 Hz, order = 424), delta (1-4 Hz; low pass filter cut-off = 1 Hz, order = 846; high pass filter cut-off = 4 Hz, order = 424), theta (4-8 Hz; low pass filter cut-off = 4 Hz, order = 424; high pass filter cut-off = 8 Hz, order = 424), and alpha bands (8-15 Hz; low pass filter cut-off = 8 Hz, order = 424; high pass filter cut-off = 15 Hz, order = 424). The power spectral density of EEG passed through the filtering processes are presented in Supplementary Figure 3.


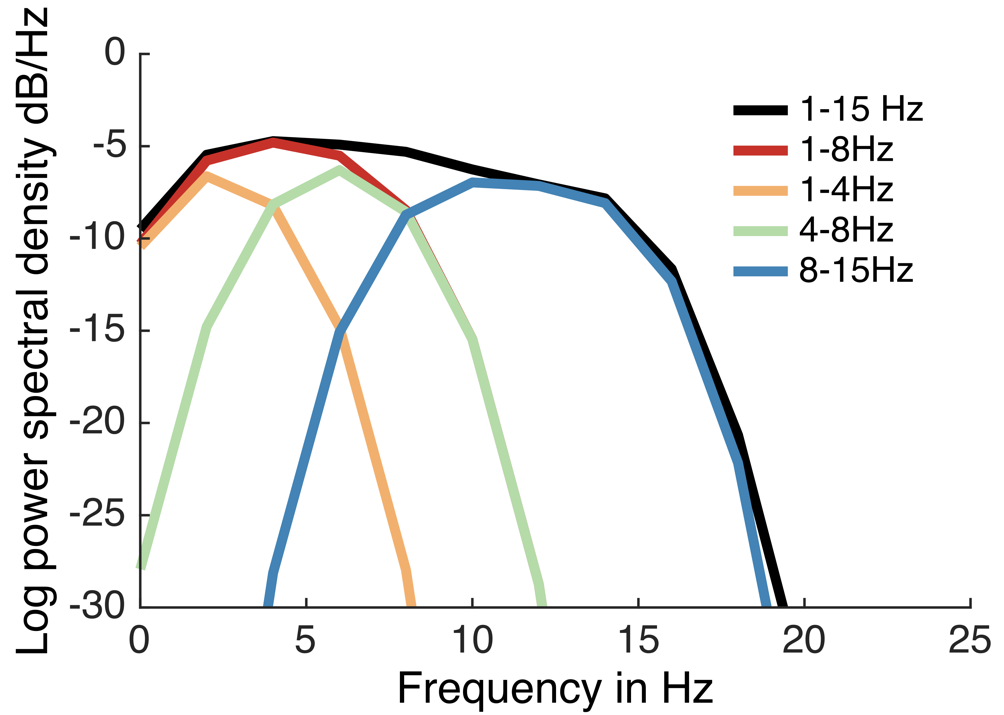


Supplementary Figure 3. Power spectral density of EEG filtered with the different bands used in this study. The data that were preprocessed using the 1-15 Hz filter were re-filtered using the different filter bands that were used in the analyses presented in this study. The power spectral density shows that the filter parameters were optimal to extract the information in the desired bands.

**Supplementary Results**

As was observed in the theta band, increased tracking for lvPPA was observed in the 1- 15 Hz band and the alpha band (8-15 Hz; Supplementary Fig 4). For the 1-15 Hz band, prediction accuracy was above chance level for all participants. There was a significant main effect of participant group [*F*(1,18)=8.20, *p*=0.01, $\eta_{G}^{2}$= 0.30]; individuals with lvPPA had higher prediction accuracies than HC. The main effect of narrative [*F*(1,18)=3.96, *p*=0.06, $\eta_{G}^{2}$ = 0.01] and the interaction [*F*(1,18)=1.91, *p*=0.18, $\eta_{G}^{2}$ = 0.005] were not significant. Previous research suggests that the information contained from 1-8 Hz reflects a linear combination of the information contained within the delta (1-4 Hz) and theta (4-8 Hz) bands (Cogan & Poeppel 2011). In the current study, we observed a strong correlation between prediction accuracies in the 1-15 Hz band and the average prediction accuracy across delta, theta and alpha bands *(r* = .90, *p* < .001)*.* It is thus likely that the main effect of participant group within the 1-15 Hz band was largely driven by differences observed in the theta band (4-8 Hz) and the alpha band (8-15 Hz; see alpha band results below).

For the alpha band (8-15 Hz), the main effects of participant group [*F*(1,18)= 9.64, *p* = 0.006, $\eta_{G}^{2}$=0.30] and narrative [*F*(1,18)=6.98, *p* = 0.02, $\eta_{G}^{2}$=0.07] were significant, but the interaction was not [*F*(1,18)=1.72, *p* = 0.21, $\eta_{G}^{2}$=0.02]. The main effects of participant group and narrative reflected a significantly larger magnitude of cortical tracking in the alpha band for individuals with lvPPA and narrative 1, respectively, although the effect size for narrative was small. However, the prediction accuracy was lower than the estimated chance level in six control participants and one individual with lvPPA. Excluding these participants caused the statistical analysis to be underpowered. Although the role of alpha cortical tracking of speech has not been thoroughly explored in the literature, stimulus-related changes in alpha band power have been linked to attention (Keitel [et al., 2014; Wöstmann et al., 2015)](https://www.zotero.org/google-docs/?PLc6qA). It may be the case that the increased magnitude of cortical tracking within the alpha band for lvPPA participants relative to HC reflects a larger allocation of attentional resources in order to successfully attend to the continuous speech stream. However, given that more than half of the HC data were not significantly different from chance in this band and that cortical tracking of speech within the alpha band is not well characterized, caution is warranted in interpreting this finding. As such, the main text focuses exclusively on cortical tracking within the delta and theta bands.


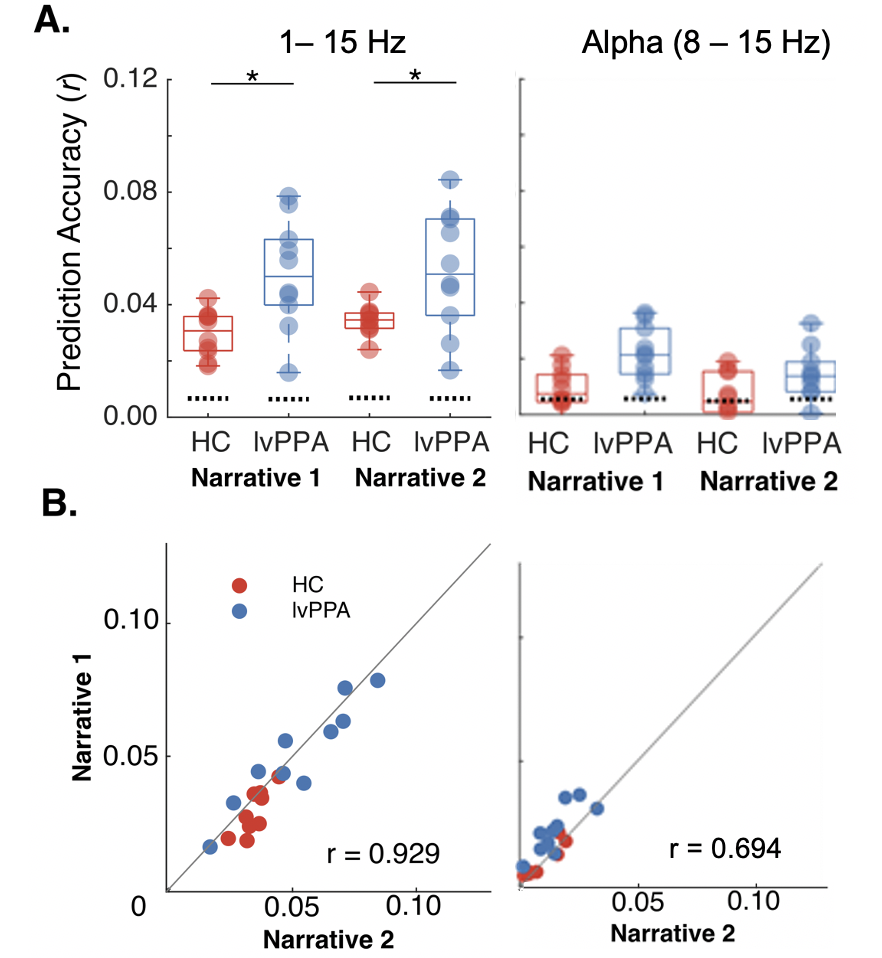


Supplementary Figure 4. Cortical tracking metrics in healthy controls (HC) and individuals with logopenic variant primary progressive aphasia (lvPPA) for the 1-15 Hz band and the alpha band (8 - 15 Hz). A. Prediction accuracies (Pearson’s *r* averaged across all EEG channels and stimulus tracks) for HC and lvPPA participants for each narrative. The dotted lines show chance levels of prediction accuracies. B. Prediction accuracies for narrative 2 plotted as a function of narrative 1. Points lying on the unity line have equal prediction accuracies for the two narratives. The plots show that the prediction accuracies for both narratives are highly correlated. Although the correlation between the two narratives was numerically larger for the 1-15 Hz band relative to the theta band presented in the main text, this difference was not statistically significant (z = -1.5, *p* = .14).

The scalp distributions of the prediction accuracies are shown in Supplementary Fig. 5. The scalp topographies are consistent with that seen in previous studies and are indicative of neurophysiologically plausible signatures. The trend of increased theta activity in the lvPPA groups is apparent in a wide range of electrodes, and thus indicates a robust neural signature.


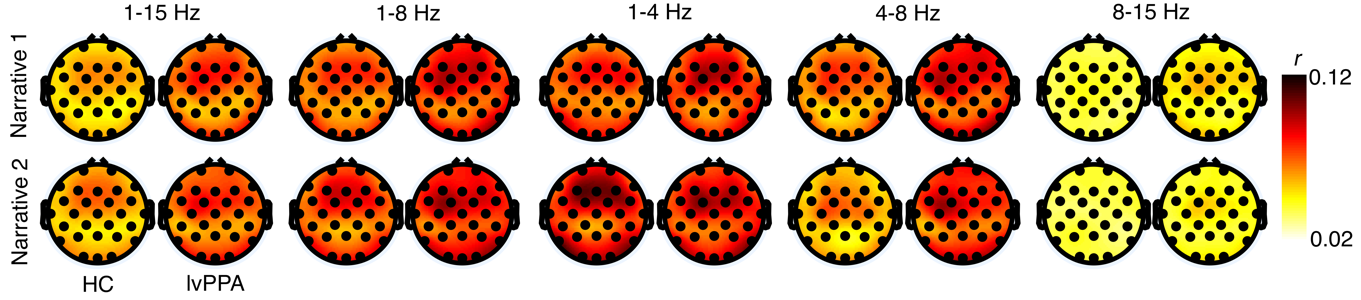
Supplementary Figure 5. Scalp distribution of the prediction accuracies in the healthy control (HC) and logopenic variant primary progressive aphasia (lvPPA) groups for both the narratives in the five different EEG bands.

**Multiple Choice Questions**

**Narrative 1 (*Alice’s Adventures in Wonderland*)**

*Correct response highlighted in yellow*

**Track 1**

Who is Alice with before she sees the white rabbit?

1.) Her cat

2.) Her friend Mabel

3.) Her sister

4.) Her mother

What color eyes did the white rabbit have?

1.) Pink

2.) Red

3.) Blue

4.) Green

**Track 2**

What does Alice pick up while falling through the well?

1.) A map

2.) An empty jar

3.) A bottle

4.) A book

What had been in the empty jar?

1.) Orange jelly

2.) Orange jam

3.) Orange honey

4.) Orange marmalade

**Track 3**

How many miles does Alice think she has fallen?

1.) 2,000

2.) 3,000

3.) 4,000

4.) 5,000

What does Alice try to do while talking to herself?

1.) Curtsey

2.) Bow

3.) Shake hands

4.) High Five

**Track 4**

Who is Dinah?

1.) Alice’s dog

2.) Alice’s bat

3.) Alice’s mouse

4.) Alice’s cat

What does Alice want to ask Dinah?

1.) Did you ever eat a rat?

2.) Did you ever eat a bat?

3.) Did you ever eat a squirrel?

4.) Did you ever eat a mouse?

**Track 5**

Where does Alice find herself after falling down the hole?

1.) A short hallway

2.) A high hallway

3.) A long hallway

4.) A dark hallway

How many legs does the little table have?

1.) Four

2.) Five

3.) Three

4.) Two

**Track 6**

What does Alice see at the end of the passage?

1.) A telescope

2.) A garden

3.) A wishing well

4.) A rose bush

What does Alice find on top of the little table the second time?

1.) A gold key

2.) A little bottle

3.) A big bottle

4.) A book of rules

**Track 7**

What was written around the neck of the bottle?

1.) ‘DRINK ME’

2.) ‘DO NOT DRINK’

3.) ‘POISON’

4.) ‘TASTE ME’

Why didn’t Alice drink from the bottle right away?

1.) She wanted to find out if the bottle was marked ‘poison’

2.) She was not able to read the label

3.) She wanted to find out who marked the bottle ‘drink me’

4.) She wanted to share the potion with the rabbit

**Track 8**

What happened to Alice after she finished the little bottle?

1.) She felt very sick

2.) She grew very large

3.) She became very small

4.) She played in the lovely garden

How does Alice try to reach the little golden key on the table?

1.) She tries to climb up one of the legs

2.) She tries to jump up to the table top

3.) She tries to drink from the little bottle

4.) She tries to knock the table down

**Track 9**

What does Alice find lying under the table?

1.) A little glass bottle

2.) A little glass box

3.) A little glass slipper

4.) A little glass jar

What happened to Alice when she ate a little bit of cake?

1.) She grew larger

2.) She grew smaller

3.) She remained the same size

4.) She began to cry

**Track 10**

What does Alice promise to give her feet every Christmas?

1.) A pair of stockings

2.) A pair of boots

3.) A pair of shoes

4.) A pair of mittens

How tall does Alice become after eating the cake?

1.) More than 6 feet

2.) More than 7 feet

3.) More than 8 feet

4.) More than 9 feet

**Track 11**

How deep was the pool of tears?

1.) 3 inches deep

2.) 4 inches deep

3.) 5 inches deep

4.) 6 inches deep

What was the White Rabbit carrying when Alice saw him?

White kid gloves and a:

1.) Waistcoat-pocket

2.) Fan

3.) Golden key

4.) Glass box

**Track 12**

What does Alice think happened to her the night before?

1.) She had food poisoning

2.) She had a nightmare

3.) She was changed into a different person

4.) She was changed into Ada

What does Alice start doing to test her past knowledge?

1.) Multiplication problems

2.) Addition problems

3.) Subtraction problems

4.) Division problems

**Track 13**

What reptile is in the lesson that Alice recites?

1.) Lizard

2.) Snake

3.) Crocodile

4.) Alligator

Who does Alice believe she has become?

1.) Ada

2.) Mabel

3.) Dinah

4.) Edie

**Track 14**

What made Alice shrink again?

1.) The fan

2.) The glass box

3.) The little bottle

4.) The white kid gloves

How tall was Alice when she measured herself?

1.) 5 ft.

2.) 4 ft.

3.) 3 ft.

4.) 2 ft.

**Track 15**

What does Alice fall in?

1.) Mineral water

2.) Spring water

3.) Salt water

4.) Lake water

Who does Alice see in the pool?

1.) A walrus

2.) A hippopotamus

3.) A cat

4.) A mouse

**Narrative 2 (*Who Was Albert Einstein?*)**

*Correct response highlighted in yellow*

**Track 1**

In what German city was Albert Einstein born?

1.) Ulm

2.) Berlin

3.) Frankfurt

4.) Weimar

What was Albert like as a child?

1.) Loud and shy

2.) Loud and aggressive

3.) Quiet and shy

4.) Quiet and aggressive

**Track 2**

What did Albert like to do as a child?

1.) Pretend to be a soldier

2.) Build houses of cards

3.) Play with other children

4.) Watch birds

Albert asked his dad and uncle about **___________**:

1.) Chemistry

2.) Planets

3.) Biology

4.) Electricity

**Track 3**

Albert thought that:

1.) Imagination was more important than knowledge

2.) Knowledge was more important than imagination

3.) Imagination was not important

4.) Knowledge is the only thing that is important

Albert was fascinated by his dad’s **_________**:

1.) Needle

2.) Compass

3.) Directions

4.) Magnet

**Track 4**

What instrument did Albert learn to play?

1.) Guitar

2.) Trumpet

3.) Flute

4.) Violin

What was Albert’s sister’s name?

1.) Emma

2.) Maya

3.) Anna

4.) Lina

**Track 5**

What did Albert expect his sister to have?

1.) Blocks

2.) A motor

3.) A hula hoop

4.) Wheels

Albert wondered if there was anything bigger than **__________**:

1.) His sister

2.) The world

3.) The universe

4.) A hill

**Track 6**

What was Albert born to be?

1.) A mechanic

2.) A thinker

3.) A veterinarian

4.) A doctor

How old was Albert when he started high school?

1.) 10

2.) 11

3.) 12

4.) 13

**Track 7**

What was Albert’s favorite subject in school?

1.) English

2.) German

3.) History

4.) Math

At home, Albert would practice **__________**:

1.) Algebra

2.) Puzzles

3.) Marching

4.) Memorizing

**Track 8**

Albert was learning about **___________**:

1.) Multiplication

2.) Division

3.) Sports

4.) Geometry

Max was Albert’s _____________:

1.) Teacher

2.) Friend

3.) Classmate

4.) Uncle

**Track 9**

Albert’s family was **___________**:

1.) Jewish

5.) Roman Catholic

6.) Muslim

7.) Buddhist

What did Albert refuse to eat?

1.) Vegetables

2.) Beef

3.) Pork

4.) Fish

**Track 10**

Where did Albert’s family move?

1.) America

2.) France

3.) Switzerland

4.) Italy

Albert’s teachers described him as _________:

1.) Highly intelligent

2.) Well behaved

3.) A lazy dog

4.) A good influence on his classmates

**Track 11**

In Italy, what did Albert like to do?

1.) Study the lives of scientists

2.) Visit history museums

3.) Visit science museums

4.) Study the lives of Italians

Which of the following is a scientist that Albert read about?

1.) Newton

2.) Mozart

3.) Galileo

4.) Aristotle

**Track 12**

While in Italy, what did Albert publish?

1.) A math textbook

2.) A scientific paper

3.) A newspaper

4.) His journal

What was Albert’s first published scientific paper about?

1.) Copernicus and Galileo

2.) The earth’s rotation around the sun

3.) Religion

4.) Electricity and magnetism

**Track 13**

What did most scientists believe existed in the empty part of space?

1.) Ether

2.) Planets

3.) Nothing

4.) Moons

What did Albert believe existed in the empty part of space?

1.) Ether

2.) Planets

3.) Nothing

4.) Moons

**Track 14**

While in Italy, Albert liked to **________**:

1.) Go sailing

2.) Hike in the mountains

3.) Visit the beach

4.) Help with his family’s business

What did Albert decide to study in college?

1.) Algebra

2.) Chemistry

3.) Physics

4.) Geometry

**Track 15**

Where did Albert go back to high school?

1.) Sweden

2.) Italy

3.) Germany

4.) Switzerland

What did Albert like to discuss with his Swiss teachers?

1.) Time

2.) Traveling to other places

3.) How much he hated his German high school

4.) His family

**Supplementary References**

Ai, H., & Lu, X. (2013). A corpus-based comparison of syntactic complexity in NNS and NS

university students’ writing. In Paul Thompson, Nicolas Ballier, & Ana Díaz Negrillo (Eds.), *Automatic treatment and analysis of learner corpus data* (249-264)*.* John Benjamins Publishing Company.

Brallier, J. (2002). *Who was Albert Einstein?* Grosset & Dunlap. Audiobook from Listening

Library, narrated by Kevin Pariseau.

Carrol, L. (1865). *Alice’s Adventures in Wonderland*. Audiobook from Librivox (Available at

https://librivox.org/alices-adventures-in-wonderland-by-lewis-carroll-5).

de Cheveigné, A., & Nelken, I. (2019). Filters: When, why, and how (not) to use them. *Neuron,*

*102*, 280-293.

Cogan, G. B., & Poeppel, D. (2011). A mutual information analysis of neural coding of speech

by low-frequency MEG phase information. *Journal of neurophysiology*, *106*(2), 554-563.

Crosse, M.J., DiLiberto, G.M., Bednar, A., & Lalor, E.C. (2016). The multivariate temporal

response function (mTRF) toolbox: A MATLAB toolbox for relating neural signals to continuous stimuli. *Frontiers in Human Neuroscience, 10*.

Davies, M. (2019). *Corpus of contemporary American English (COCA)*. (Available at english-

corpora.org/coca).

Delorme, A., & Makeig, S. (2004). EEGLAB: An open source toolbox for analysis of single-trial

EEG dynamics including independent component analysis. *Journal of Neuroscience Methods, 134*, 9-21.

DiLiberto, G.M., Cross, M.J., & Lalor, E.C. (2018). Cortical measures of phoneme-level speech

encoding correlate with the perceived clarity of natural speech. *Eneuro, 5*(2).

DiLiberto, G.M., O’Sullivan, J.A., & Lalor, E.C. (2015). Low-frequency cortical entrainment to

speech reflects phoneme-level processing. *Current Biology, 25*, 2457-2465.

Ding, N., Patel, A., Chen, L., Butler, H., Luo, C., & Poeppel, D. (2017). Temporal modulations

in speech and music. *Neuroscience & Biobehavioral Reviews, 81*, 181-187.

Hamilton, L.S., Edwards, E., & Chang, E.F. (2018). A spatial map of onset and sustained

responses to speech in the human superior temporal gyrus. *Current Biology, 28*, 1860-1871.

Keitel, C., Quigley, C., & Ruhnau, P. (2014). Stimulus-driven brain oscillations in the alpha

range: Entrainment of intrinsic rhythms or frequency-following-response? *Journal of Neuroscience, 34*, 10137-10140.

Lu, X. (2010). Automatic analysis of syntactic complexity in second language writing.

*International Journal of Corpus Linguistics, 15*, 474-496.

Lu, X. (2011). A corpus-based evaluation of syntactic complexity measures as indices of college-

level ESL writers’ language development. *TESOL Quarterly, 45*, 36-62.

Lu, X., & Ai, H. (2015). Syntactic complexity in college-level English writing: Differences

among writers with diverse L1 backgrounds. *Journal of Second Language Writing, 29*, 16-27.

Maris, E., & Oostenveld, R. (2007). Nonparametric statistical testing of EEG- and MEG-data.

*Journal of Neuroscience Methods, 164*, 177-190.

Mitchell, J. (2013, March). Learning semantic representations in a bigram language model.

In *Proceedings of the 10th International Conference on Computational Semantics (IWCS 2013)–Short Papers* (pp. 362-368).

Mullen, T. R., Kothe, C. A., Chi, Y. M., Ojeda, A., Kerth, T., Makeig, S., ... & Cauwenberghs,

G. (2015). Real-time neuroimaging and cognitive monitoring using wearable dry EEG. *IEEE Transactions on Biomedical Engineering*, *62*(11), 2553-2567.

Schneider, W., Eschman, A., & Zuccolotto, A. (2002). *E-Prime: User's guide*. Psychology

Software Incorporated.

Slaney, M. (1998). Auditory toolbox. *Interval Research Corporation, Tech. Rep*, *10*, 1194.

Vanthornhout, J., Decruy, L., Wouters, J., Simon, J. Z., & Francart, T. (2018). Speech

intelligibility predicted from neural entrainment of the speech envelope. *Journal of the Association for Research in Otolaryngology*, *19*(2), 181-191.

Wilson, S. M., Yen, M., & Eriksson, D. K. (2018). An adaptive semantic matching paradigm for

reliable and valid language mapping in individuals with aphasia. *Human Brain Mapping*, *39*(8), 3285-3307.

Wöstmann, M., Herrmann, B., Wilsch, A., & Obleser, J. (2015). Neural alpha dynamics in

younger and older listeners reflect acoustic challenges and predictive benefits. *Journal of Neuroscience*, *35*(4), 1458-1467.
